# Oncolytic Activity of a Recombinant Measles Virus, Blind to Signaling Lymphocyte Activation Molecule, Against Colorectal Cancer Cells

**DOI:** 10.1038/srep24572

**Published:** 2016-04-19

**Authors:** Yosuke Amagai, Tomoko Fujiyuki, Misako Yoneda, Koichiro Shoji, Yoichi Furukawa, Hiroki Sato, Chieko Kai

**Affiliations:** 1 Laboratory Animal Research Center, The Institute of Medical Science, The University of Tokyo, Tokyo, Japan; 2 Division of Clinical Genome Research, The Institute of Medical Science, The University of Tokyo, Tokyo, Japan

## Abstract

Oncolytic virotherapy is a distinctive antitumor therapy based on the cancer-cell-specific infectivity and killing activity of viruses, which exert a considerable antitumor effect with only a few treatments. Because colorectal cancer cells often acquire resistance to the molecular-targeted therapies and alternative treatments are called for, in this study, we evaluated the oncolytic activity against colorectal cancer cells of a recombinant measles virus (rMV-SLAMblind), which is blind to signaling lymphocytic activation molecule (SLAM) and infects target cells via nectin-4/poliovirus receptor-related 4 protein. We examined 10 cell lines including 8 cell lines that were resistant to epidermal-growth-factor-receptor (EGFR) targeted therapy. rMV-SLAMblind infected and lysed the nectin-4-positive cell lines dependently on nectin-4 expression, in spite of mutation in EGFR cascade. Tumour progression in xenograft models was also abrogated by the virus, and the infection of cancer cells *in vivo* by the virus was demonstrated with both flow cytometry and a histological analysis. Therefore, rMV-SLAMblind is considered a novel therapeutic agent for colorectal cancers, including those resistant to molecular-targeted therapies.

Colorectal cancer is one of the most commonly diagnosed malignancies throughout the world with a high mortality rate^1^. Approximately 25% of patients with colorectal cancer display metastatic disease. Various kinds of molecular-targeted agents, including biopharmaceutical products such as antigen-specific antibodies have been used to treat colorectal cancers, and epidermal growth factor receptor (EGFR) is one of the major targets of these treatments because >80% of these tumours express EGFR^2^,^3^,^4^. The superiority of molecular-targeted inhibitors is their high specificity and lower toxicity than those of conventional chemotherapeutic agents. However, accumulating evidence indicates that the therapeutic outcomes after treatment with these agents depend on the mutational status of the target molecules in each tumour. In particular, mutations of either the Kirsten rat sarcoma viral oncogene homolog (*KRAS*), v-*raf* murine sarcoma viral oncogene homolog B1 (*BRAF*), or phosphatidylinositol-4,5-bisphosphate 3-kinase, catalytic subunit alpha (*PIK3CA*) gene, which encode the molecules that are activated downstream from EGFR, cause cells to acquire resistance to anti-EGFR antibodies, such as bevacizumab, cetuximab and panitumumab^5^,^6^,^7^,^8^,^9^. Moreover, although mutations in either *KRAS* or *PIK3CA* occur in approximately 50% of all patients with colorectal cancer^6^,^7^,^8^,^9^, no alternative molecular-targeted approach has been developed to eradicate these mutation-positive tumours.

Oncolytic virotherapy is a promising approach to the eradication of cancers^10^,^11^, because it takes advantage of the natural or acquired characteristics of a virus to target cancer cells^10^,^11^. Reovirus and Newcastle disease virus, for example, have a natural preference for cancer cells, whereas others, such as adenovirus, herpes simplex virus, and vesicular stomatitis virus have been genetically modified to confer greater infectivity and a greater replication capacity in tumour cells than in non-tumour cells^10^,^11^,^12^. We recently demonstrated that genetically modified recombinant measles virus (rMV), which is derived from a wild-type MV (HL strain) but is blind to the signaling lymphocyte activation molecule (SLAM/CD150) protein (rMV-SLAMblind), selectively infected and killed breast cancer cells in a nectin-4/poliovirus receptor-related 4-dependent manner^13^. Both SLAM and nectin-4 have been shown to be MV receptors^14^,^15^,^16^. SLAM expression is observed in a wide range of immune cells^17^, and the pathogenesis of wild-type MV is mediated by the infection of immune cells via SLAM. Nectin-4 expression in the normal human body is observed in the placenta and is slightly detected in the epithelial cells of the trachea, where it forms adherens junctions together with E-cadherin^17^,^18^,^19^. rMV-SLAMblind caused no pathogenicity in rhesus or cynomolgus monkeys^13^. Recently, Noyce *et al*.^16^ reported that some colorectal adenocarcinoma cell lines express nectin-4. In this study, we examined the antitumor effects of rMV-SLAMblind on colorectal cancer cells to investigate whether rMV-SLAMblind is an effective agent for treatment of colorectal cancer, especially with resistance to molecular targeted therapies.

## Results

### Nectin-4 expression in colorectal cancer cell lines

**Figure 1:**
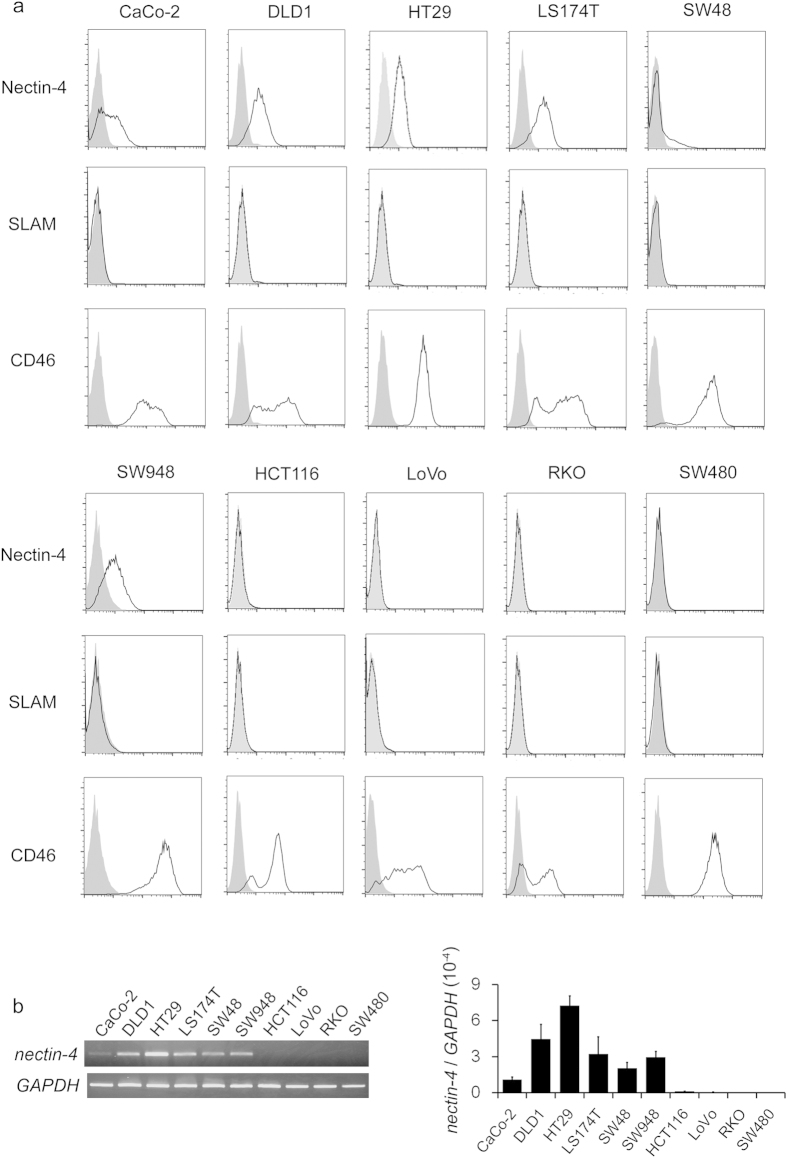
Expression of MV receptors on colorectal cancer cells. (**a**) Flow-cytometric analysis of cell-surface proteins that are associated with MV entry. A total number of 10^6^ cells were stained with each primary and secondary Ab (black line). For nectin-4 detection, mAb (clone N4.61) was used in this experiment. The grey histogram indicates the IgG control for each cell line. Shown are representative data of three independent experiments. (**b**) RT-PCR analysis of each colorectal cancer cell line. Representative data of the electrophoresis (left) as well as relative expression level of *nectin-4* based on the qRT-PCR analysis (right) is presented. In the qRT-PCR analysis, expression level of *nectin-4* was normalized to *GAPDH*, and the average ± SD of three independent experiments is indicated.

A flow-cytometric analysis was conducted to examine the expression of nectin-4 in colorectal cancer cell lines. Among the 10 cell lines examined (CaCo-2, DLD1, HT29, LS174T, SW48, SW948, HCT116, LoVo, RKO, and SW480), the CaCo-2, DLD1, HT29, LS174T, SW48, and SW948 cell lines expressed nectin-4, whereas the others did not (Fig. 1a, Table 1). Among these, nectin-4 expression in SW48 cells was heterogeneous, of which approximately 15% cells only express nectin-4 on the cell surface (Fig. 1a). The wild-type MV strains, including the HL strain, use SLAM as their receptor, whereas MV vaccine strains use CD46^14^,^20^, which is a recognition molecule expressed ubiquitously in human nucleated cells. We also analysed the expression of these receptors and observed that all the cell lines tested were negative for SLAM and positive for CD46 (Fig. 1a). To ascertain the expression of *nectin-4* at the messenger RNA (mRNA) level, reverse transcription and polymerase chain reaction (RT-PCR) were performed. Higher expression of *nectin-4* mRNA was observed in the cells that were positive for nectin-4 in the flow-cytometric analysis than in those that were nectin-4-negative on flow cytometry (Fig. 1a,b). Regarding SW48 cells, *nectin-4* mRNA expression was as high as other nectin-4-positive cells in spite of their heterogeneous nectin-4 expression (Fig. 1b).

### Infectivity and cytotoxicity of rMV-SLAMblind in colorectal cancer cell lines

**Figure 2:**
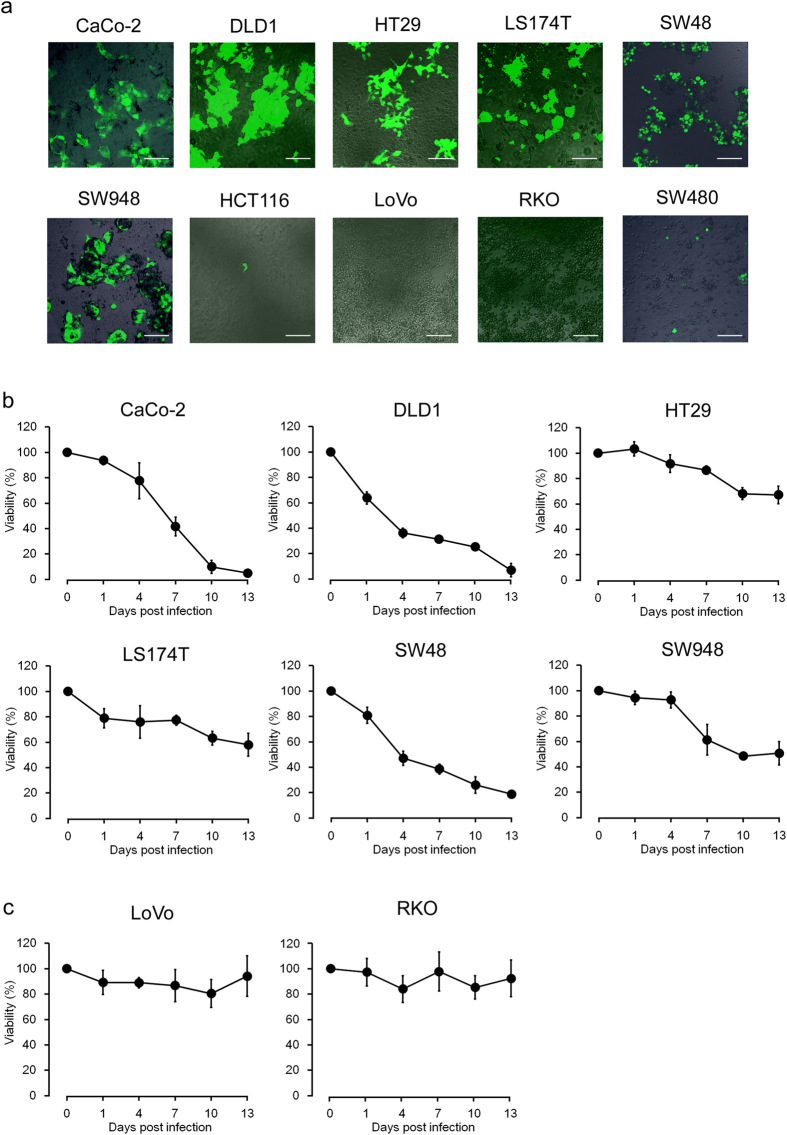
Infectivity and killing activity of rMV-EGFP-SLAMblind in colorectal cancer cells. (**a**) Representative fluorescence microscopy data. Cells were inoculated with rMV-EGFP-SLAMblind at MOI 2 and incubated for 72 h. Fluorescence microscopy was used to detect infection with rMV-EGFP-SLAMblind. Shown are representative data of three independent experiments. Original magnification, 20× objective lens. Bar, 100 μm. Changes in cell viability in nectin-4-positive (**b**) and nectin-4-negative (**c**) colorectal cancer cells. Cells were inoculated with rMV-EGFP-SLAMblind at MOI 2, and a WST assay was conducted on the indicated dpi. Each datum represents the mean ± SD of three independent experiments.

To investigate the susceptibility of the colorectal cancer cells to rMV-SLAMblind, each cell line was inoculated with the virus at a multiplicity of infection (MOI) of 2 and examined with fluorescence microscopy at 3 days post-infection (dpi). To visualize viral infection, enhanced green fluorescent protein (EGFP)-expressing rMV-SLAMblind (rMV-EGFP-SLAMblind) was used, based on the previous observations that the insertion of *EGFP* does not affect the growth kinetics of rMVs^21^,^22^. As shown in Fig. 2a, the replication of rMV-EGFP-SLAMblind was only observed in the nectin-4-positive cells. A water-soluble tetrazolium salt (WST) assay was performed to determine the killing activity of rMV-EGFP-SLAMblind in nectin-4-positive colorectal cancer cell lines. The inoculation of nectin-4-positive cells with rMV-EGFP-SLAMblind caused a time-dependent reduction in cell viability compared with that of the control (Fig. 2b). In contrast, the viabilities of nectin-4-negative cells were not altered after their inoculation with rMV-EGFP-SLAMblind (Fig. 2c).

### Antitumor effects of rMV-SLAMblind *in vivo*

**Figure 3:**
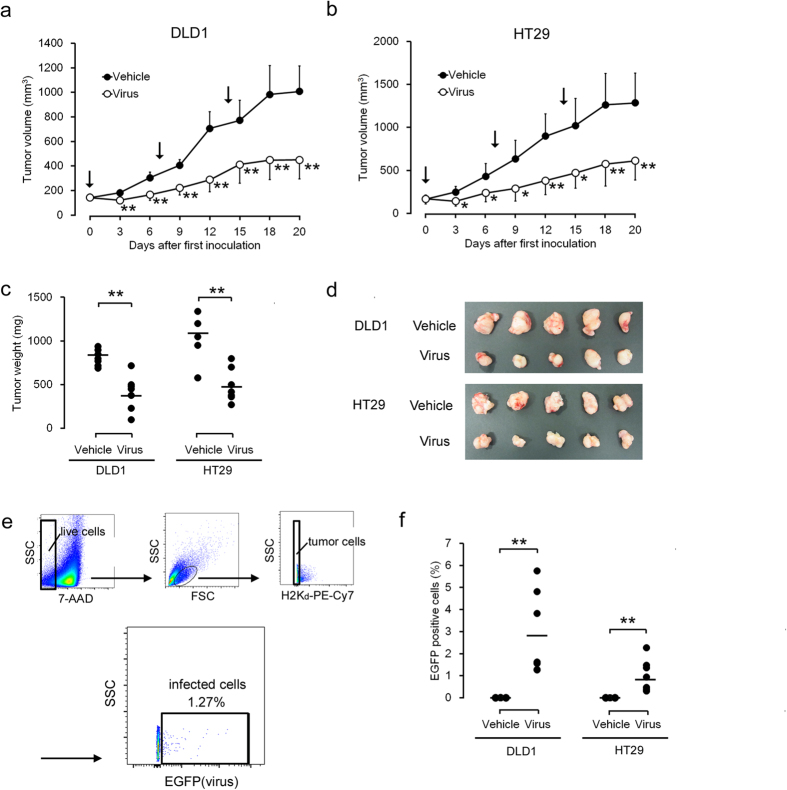
Antitumor effects of rMV-EGFP-SLAMblind *in vivo*. Growth curves of DLD1 (**a**) and HT29 cells (**b**) *in vivo*. A total number of 10^6^ DLD1 and HT29 cells were injected subcutaneously into seven SCID mice each, and the tumour sizes were measured every 2 or 3 days. Arrows indicate the days upon which the mice were injected intratumorally with rMV-EGFP-SLAMblind at 10^6^ TCID_50_/mouse (days 0, 7, and 14). Each datum represents a mean ± SD (*n* = 7). *p < 0.05, **p < 0.01 compared with the vehicle-treated control on Welch’s *t* test. (**c**) Antitumor effects conferred by rMV-EGFP-SLAMblind. All mice were euthanized on day 20 and their tumours weighed. The bar indicates the mean average for each group. (*n* = 7). **p < 0.01 compared with the vehicle-treated controls on Welch’s *t* test. (**d**) Representative photographs of a tumour in each treatment group. (**e**) Flow-cytometric analysis of live virus-infected tumour cells. All plots are density plots and fluorescent intensities were plotted on bi-exponential axes. (**f**) The plots indicate the proportion of EGFP-positive cells in each mouse (*n* = 7 in each group), and the bar indicates the mean for each group. **p < 0.01 compared with the vehicle-treated control on Welch’s *t* test.

**Figure 4:**
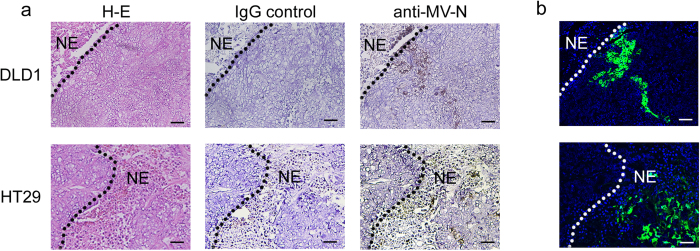
Histological analysis of tumours established *in vivo*. (**a**) Immunohistochemical analysis and hematoxylin-eosin (H-E) staining of tumours *in vivo*. DLD1 or HT29 cells grown and treated with rMV-EGFP-SLAMblind *in vivo* were stained with anti-MV-N protein Ab. NE indicates necrotic region. Original magnification, 20× objective lens. Bar; 100 μm. (**b**) Observation of tumour tissues under a fluorescence microscope. The presence of rMV-EGFP-SLAMblind was detected as virus-derived EGFP fluorescence. NE indicates necrotic region. Original magnification, 20× objective lens. Individual panels for each tumour show serial sections.

The antitumor effects of rMV-EGFP-SLAMblind *in vivo* were examined using xenograft models. DLD1 and HT29 cells were transplanted into C.B-17/Icr-*scid/scid*Jcl (SCID) mice. When the tumours reached 200 mm^3^, they were inoculated with rMV-EGFP-SLAMblind three times at weekly intervals. As shown in Fig. 3a, the administration of rMV-EGFP-SLAMblind exerted potent antitumor effects on the DLD1 cells, resulting in a reduction in the tumour volume of approximately 55% compared with the control. Similar results were obtained with the HT29 cell transplantation model, resulting in a reduction in the tumour volume of approximately 60% compared with the control (Fig. 3b). Twenty days after the first inoculation, each mouse was euthanized and their tumours weighed. The mean tumour weight in the virus-treated group was significantly lower than that in the control group for each type of tumour (Fig. 3c,d). We also performed a flow-cytometric analysis to investigate whether rMV-EGFP-SLAMblind remained within the tumour a week after the last administration of the virus. The live cell population was selected based on the incorporation of 7-amino-actinomycin D (7-AAD), detected with forward/side scatter (FSC/SSC; Fig. 3e). The mouse-derived H2K_d_-positive cells were gated out to focus on the live tumour cells (Fig. 3e). The proportion of EGFP-positive cells within the gate, which represented the rMV-EGFP-SLAMblind-infected live tumour cells, was 1–6% (2.9% on average) in the DLD1 cells and 0.2–2% (1.0% on average) in the HT29 cells (Fig. 3e,f). In contrast, the tumours in the control group were negative for EGFP (Fig. 3f). To analyse the distribution of the virus-infected cells, a histopathological analysis was performed on tumour tissues treated with rMV-EGFP-SLAMblind. Large necrotic regions were observed histopathologically in the tumour masses from both xenograft models, established with DLD1 and HT29 cells (Fig. 4a, H–E). Positive EGFP fluorescence, indicating the presence of the virus in growing cells, was observed in the area adjacent to the necrotic region (Fig. 4b). MV-N protein was immunostained in an analysis of serial sections, and the MV-N-positive area corresponded to the necrotic region and the EGFP-positive area in both xenograft models (DLD1 and HT29 cells) (Fig. 4a,b).

### Unique pattern of nectin-4 expression in SW48 cells

**Figure 5:**
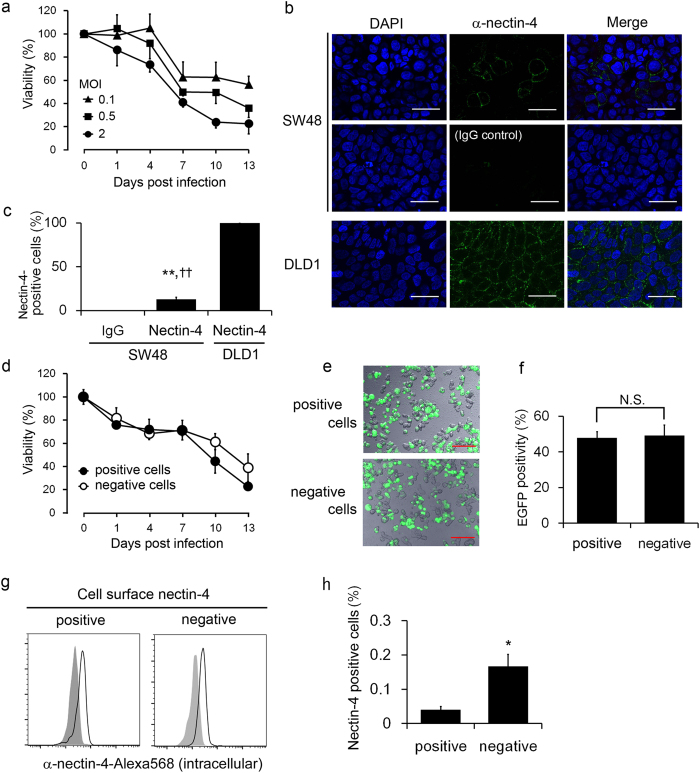
Unique nectin-4 expression in SW48 cells. (**a**) Changes in cell viability in SW48 cells after rMV-EGFP-SLAMblind inoculation at different MOIs. A WST assay was conducted on the indicated dpi. Each datum represents the mean ± SD of three independent experiments. (**b**) Immunocytochemical analysis of SW48 cells. Cells were stained with anti-nectin-4 pAb, followed by Alexa-Fluor-488-conjugated anti-goat IgG Ab. The cells were then fixed, stained with Hoechst 33342, and observed under a fluorescence microscope. Isotype IgG was used as the negative control. Shown are representative data of three independent experiments. Original magnification, 60× objective lens. Bar, 30 μm. (**c**) The graph shows the proportions of Alexa-Fluor-488-positive cells as the means ± SD of five randomly selected microscopic fields. **^,††^*p* < 0.01 compared with the isotype-IgG-stained control and DLD1 cells on Welch’s *t* test, respectively. (**d**) WST assay of sorted SW48 cells. A WST assay was conducted using either nectin-4-positive or nectin-4-negative cells after cell sorting. (**e**) Representative fluorescence microscopic data of three independent experiments. Cells were inoculated with rMV-EGFP-SLAMblind at MOI 2 for 72 h. Original magnification, 20× objective lens. Bar, 100 μm. (**f**) The graph shows the proportions of EGFP-positive cells of five randomly selected microscopic fields. The values are the means ± SD of three independent experiments. N.S., not significant on Welch’s *t* test. (**g**) Flow-cytometric analysis of intracellular nectin-4 in SW48 cells. Cell-surface and intracellular nectin-4 expression was detected with mouse anti-nectin-4 mAb (clone N4.61) and goat anti-nectin-4 pAb, respectively. Each histogram indicates the intracellular nectin-4 level in either the nectin-4-positive or nectin-4-negative population. The grey histogram indicates the IgG control for each fraction. Shown are representative data of three independent experiments. (**h**) Flow-cytometric analysis of cell-surface nectin-4 expression in a short-term culture of SW48 cells. Nectin-4-negative SW48 cells were sorted and cultured in FBS-containing medium for 2 h, and the gain of surface nectin-4 expression was examined. The negative control cells were stained with nectin-4 Ab without culture. The data represent the means ± SD of the nectin-4-positive cells in three independent experiments. *p < 0.05 on Welch’s *t* test.

The results described above clearly demonstrate the nectin-4-dependent infectivity and killing activity of rMV-EGFP-SLAMblind against colorectal cancer cells. Interestingly, the virus showed strong killing activity against SW48 cells (Fig. 2b), even though only approximately 15% of the population expressed nectin-4 in SW48 cells (Fig. 1a). To confirm lower MOI efficiently kills such cells with low expression of nectin-4, we inoculated the cells with three MOIs (0.1, 0.5, and 2) and found that, even at a lowest MOI, SW48 was killed efficiently (Fig. 5a). The heterogeneity of nectin-4 expression on SW48 cells was also confirmed with an immunofluorescence assay (Fig. 5b,c). To analyse whether rMV-EGFP-SLAMblind infects only the nectin-4-positive sub-population of SW48 cells, the nectin-4-positive and nectin-4-negative populations were sorted, and a WST assay was performed. The purity of either nectin-4-positive or -negative cells was >99% respectively. Surprisingly, not only the sorted nectin-4-positive single cells but also the nectin-4-negative cell population were infected with the virus, and were killed by it to the same extent (Fig. 5d,e,f). Based on the nectin-4-dependent infectivity of rMV-EGFP-SLAMblind in other cell lines, we hypothesized that SW48 cells constitutively express intracellular nectin-4 and intermittently express it on the cell surface, although most of the protein localizes in the cytoplasm. To examine this possibility, the total nectin-4 expression (both intracellular and cell-surface expression) and its cell-surface expression were determined. Live cells were stained with a mouse anti-nectin-4 monoclonal antibody (mAb) and an anti-mouse secondary Ab, fixed, permeabilized, and then stained with goat anti-nectin-4 polyclonal Ab (pAb), followed by an anti-goat secondary Ab. As expected, the total expression of nectin-4 was detected, regardless of the surface expression of nectin-4 (Fig. 5g). To directly determine whether nectin-4-negative cells become nectin-4-positive cells, the nectin-4-negative fraction of SW48 cells was sorted and cultured in foetal bovine serum (FBS)-containing medium for 2 h, and the extracellular nectin-4 expression was reanalysed. As shown in Fig. 5h, the population of cells expressing surface nectin-4 increased after culture. To examine whether this unique nectin-4 expression pattern in SW48 cells is attributable to the amino acid sequence of nectin-4, a sequence analysis was performed with mRNA obtained from SW48 cells. However, the sequence of the *nectin-4*-coding region, including its signal peptide, was identical to that in the GenBank database (accession number NM_030916.2) and no cell-line-specific mutation was observed (data not shown).

## Discussion

In this study, we have demonstrated the antitumor effects of rMV-SLAMblind on colorectal cancer cells both *in vitro* and *in vivo*. Nectin-4 expression was observed in six of the 10 cancer cell lines tested. Targeted cancer therapy is one of the major treatments currently used for cancer. Resistance to EGFR inhibitors, which are among the most commonly used targeted therapies for colorectal cancer, occurs with mutations in EGFR-related molecules. Eight of the 10 tested cell lines had various mutations in the *KRAS*, *BRAF*, and/or *PI3KCA* oncogenes (Table 1), which confer resistance to anti-EGFR therapies^5^,^6^,^7^,^8^,^9^. Half of these cell lines (four of eight) expressed nectin-4 and were infected and killed by rMV-SLAMblind in this study. In addition, rMV-SLAMblind showed antitumor effect in xenograft models, even of HT29 to which cytotoxicity of rMV-SLAMblind *in vitro* was not high. These results suggest that rMV-SLAMblind is a novel therapeutic tool for the treatment of nectin-4-positive colorectal cancers, including those that are refractory to molecular-targeted therapies. Interestingly, serum nectin-4 levels have been used as prognostic markers in breast, ovary, and lung cancer^19^,^23^,^24^,^25^. Moreover, because nectin-4 boosts the anchorage-independent growth of epithelial cells^26^, treatment with rMV-SLAMblind may reduce the number of cancer cells with a malignant phenotype, which drive cancer invasion and metastasis.

One of the therapeutic strengths of oncolytic viruses is their capacity to replicate within tumour cells. Theoretically, the number of oncolytic viral particles can increase beyond the number initially injected, exerting a relatively long-lasting antitumor effect after one treatment. The data from both our flow-cytometric and histological analyses exemplify this, demonstrating the presence of rMV-SLAMblind within the tumour cells *in vivo* at least 7 days in this study. MV is also known to induce strong cell-mediated immune responses^27^, which target MV-infected cells. Therefore, rMV-SLAMblind-infected cells should be the targets of the cell-mediated immunity of the host and will also be killed by it. Adequate *in vivo* models using immunocompetent animals must be established to predict more accurately the clinical efficacy of rMV-SLAMblind in cancer patients, and to understand the involvement of the host immune responses in virotherapies.

Interestingly, the nectin-4 expression pattern was distinctive in SW48 cells. Our data suggest that nectin-4 is constitutively expressed intracellularly and its surface expression is rapidly turned over in SW48 cells. Because no SW48-cell-line-specific mutation in the *nectin-4* mRNA was detected, including in the region coding the signal peptide, a further investigation is necessary to clarify the mechanism of this nectin-4 turnover. In addition, SW48 cells were efficiently killed by rMV-SLAMblind. This raises the possibility that rMV-SLAMblind is even effective in some tumours that express nectin-4 intermittently. On the other hand, it was reported that there is a cell line that was partially nectin-4-positive but not susceptible to MV infection (SCaBER, urinary bladder squamous cell line)^15^, and that nectin-4 is necessarily complexed with afadin to work as entry receptor for MV^28^. Thus, effects of expression level of nectin-4 associated proteins on susceptibility of cancer cells to rMV-SLAMblind should be investigated in future.

In conclusion, we have demonstrated the antitumor effects of rMV-SLAMblind against nectin-4-positive colorectal cancer cells, including cells resistant to EGFR inhibitors, indicating that it is a potential novel therapy for colorectal cancers.

## Materials and Methods

### Cell culture

All colorectal cancer cell lines, which expressed various kinds of *KRAS*, *BRAF* and *PIK3CA* status^29^,^30^ (Table 1), were obtained from the American Type Culture Collection (Manassas, VA)^31^. MCF7 human breast cancer cells were obtained from the RIKEN Cell Bank (Tsukuba, Japan). DLD1, HT29, and SW48 cells were cultured in RPMI1640 medium (Life Technologies, Gaithersburg, MD) supplemented with 10% FBS (AFC Biosciences, Lenexa, KS) and antibiotics. All other cells, were maintained in Dulbecco’s modified Eagle’s medium (Life Technologies) supplemented with 10% FBS and antibiotics.

### RT-PCR and sequence analysis

Cells were lysed with TRIzol LS Reagent (Life Technologies) and total RNA was extracted according to the manufacture’s instruction. Complementary DNA (cDNA) was synthesized with an RT-PCR kit (PrimeScript; Takara, Shiga, Japan). PCR amplification of human *nectin-4* and glyceraldehyde-3-phosphate dehydrogenase (*GAPDH*) was performed with AmpliTaq polymerase (Life Technologies) and specific primers: *nectin-4*-specific forward primer 5′-ACATCCTCCACGTGTCCTTC-3′, *nectin-4*-specific reverse primer 5′-CAAAGTGTCCCCATCCACTC-3′; *GAPDH*-specific forward primer 5′-CACCCACTCCTCCACCTTTGAC-3′, and *GAPDH*-specific reverse primer 5′-GTCCACCACCCTGTTGCTGTAG-3′. The PCR condition for amplifying *nectin-4* cDNA was as follows; 95 °C for 10 min + (95 °C for 30 sec + 50 °C for 30 sec + 72 °C for 30 sec) × 30 cycles + 72 °C for 10 min. For amplifying *GAPDH* cDNA, PCR cycling was modified to the following conditions; 95 °C for 10 min + (95 °C for 30 sec + 52 °C for 30 sec + 72 °C for 30 sec) × 25 cycles + 72 °C for 10 min. qRT-PCR was performed with QuantStudio 3 (Life Technologies) using SYBR Premix Ex Taq II kit (Takara) in the presence of each primer described above.

To determine the whole coding region sequence of *nectin-4*, PCR was conducted with *LA Taq* DNA Polymerase (Takara) and the following primer set: forward primer 5′-GGTCAGTTCCTTATTCAAGTCTGC-3′ and reverse primer 5′-GCTAAAATCTCCCATGTCAACAG-3′. The PCR products were cloned into a TA cloning vector (pGEM-T; Promega, Madison, WI), and then sequenced on an ABI 3130 Genetic Analyzer (Life Technologies). The sequence was compared with the GenBank reference (accession number NM_030916.2).

### Flow cytometry

A total number of 10^6^ cells were labelled with 0.5 μg of primary Ab in 100 μL of Hank’s balanced salt solution (HBSS; Life Technologies) containing 2% FBS and 5 mM 4-(2-hydroxyethyl)-1-piperazine ethanesulfonic acid (HEPES), and then with an Alexa-Fluor-488-conjugated anti-mouse IgG Ab (Life Technologies) diluted 1:500. The following Abs were used as primary Abs: anti-human SLAM mAb (clone 7D4; BioLegend, San Diego, CA), anti-human CD46 mAb (clone M177; Hycult Biotech, Uden, Netherlands), anti-nectin-4 mAb (clone N4.61; Millipore, Billerica, MA), and mouse control IgG (R&D Systems, Minneapolis, MN). To detect intracellular nectin-4, cells were stained with an anti-nectin-4 mAb and Alexa-Fluor-488-conjugated anti-mouse IgG Ab. The cells were then fixed with 4% paraformaldehyde (PFA), permeabilized with 1% Triton X-100, and stained with goat anti-nectin-4 pAb and Alexa-Fluor-568-conjugated anti-goat IgG Ab. To exclude the dead cells, either 7-AAD (Beckman Coulter, Tokyo, Japan) or 4′,6-diamidino-2-phenylindole (DAPI; Dojindo, Kumamoto, Japan) was used in all analyses. Analyses were carried out with a BD FACSCalibur flow cytometer (BD Biosciences, San Diego, CA) or BD FACSVerse flow cytometer (BD Biosciences), and the data were processed with the FlowJo FACS analysis software ver. 9.5.3 (Tree Star, Inc., Ashland, OR).

### Immunocytochemistry

A total number of 10^6^ cells were labelled with 0.5 μg of anti-nectin-4 mAb, Alexa-Fluor-488-conjugated anti-mouse IgG Ab diluted 1:500 and DAPI. The cells were then mounted in Dako Glycerol Mounting Medium (Dako, Glostrup, Denmark) and positive reactions were detected with a FV1000 microscope and FV10-ASW software ver. 02.01. (Olympus Japan, Tokyo, Japan). Isotype mouse IgG was used as the negative control.

### Virus

rMV-EGFP-SLAMblind were grown in MCF-7 as described previously^13^, and the virus stocks were kept at −70 °C. The titres of rMV-EGFP-SLAMblind were determined as 50% tissue culture infectious doses (TCID_50_) in MCF7 cells based on the Reed–Muench method^32^. Briefly, MCF-7 cells in 96-well plates were inoculated with virus suspensions, which were serially 10-fold diluted. Then plates were incubated for 7 days and viral titres were determined. All MOIs used in this study were determined based on this value. The titres calculated using MCF7 cells were almost identical to the one using nectin-4-expressing Vero cells (data not shown).

### Viral infection of each colorectal cancer cell line with rMV-EGFP-SLAMblind

Monolayers of cells in 96-well plates were infected with rMV-EGFP-SLAMblind at MOI 2 and the EGFP expression in the cells was detected at 3 dpi with an FV1000 microscope.

### WST assay

Cell viability was determined with the WST-1 Cell Proliferation Kit (Takara), according to the manufacturer’s instructions. Briefly, 0.5–2 × 10^4^ cells in 96-well plates were infected with rMV-EGFP-SLAMblind and cultured for the indicated days in FBS-containing medium. For the evaluation of cell viability, 10 μl WST-1 solution was added in each well, incubated for 2–4 h, and then the absorbance at 450 nm was measured at the indicated dpi using a Model 450 Microplate Reader (Bio-Rad, Hercules, CA). The viability of the cells was determined as described previously^13^.

### Antitumor effects of rMV-SLAMblind *in vivo*

All experiments with animals both complied with the standards specified in the guidelines of the Experimental Animal Committee of The University of Tokyo, and were reviewed and approved by the institutional committee. A total number of 5 × 10^6^ cells were suspended in a 50% concentration of growth factor-reduced Matrigel (BD Biosciences) and injected subcutaneously into the right flanks of 6-week-old female C.B-17/Icr-*scid/scid*Jcl mice (Clea Japan, Tokyo, Japan). The mice were carefully monitored for the development of palpable or visible tumours at the sites of injection. The tumours were measured with a calliper every 2 or 3 days. The tumour volume (V) was calculated with the formula V = ab^2^/2, where a and b are the length and width of the tumour mass (in mm), respectively. The mice were administered 10^6^ TCID_50_ of rMV-EGFP-SLAMblind or plain RPMI 1640 medium intratumorally three times at weekly intervals. Twenty days after the first inoculation, the mice were killed and the tumour tissues analysed immunohistochemically.

### Flow-cytometric analysis of *in vivo* tumours

Tumour tissues were digested with HBSS containing 5 mM HEPES, 2% FBS, 1 mg/mL collagenase (Wako Pure Chemical Industries, Osaka, Japan) and 0.1% DNase I (Life Technologies). The cells were stained with 7-AAD and phycoerythrin–Cy7-conjugated anti-mouse H2K_d_ Ab (clone SF 1–1.1; BD Biosciences), and then fixed with 4% PFA. The cells were analysed with a BD FACSVerse flow cytometer, and the data were processed using the FlowJo FACS analysis software ver. 9.5.3.

### Immunohistochemistry and fluorescence microscopy

Tumour tissues were fixed in 4% PFA overnight at 4 °C, embedded in Tissue-Tek OCT compound (Sakura Finetechnical Co., Ltd, Tokyo, Japan), and frozen in liquid nitrogen. The tissues were cut into 6 μm thick sections, fixed in acetone, washed in phosphate-buffered saline, and blocked with hydrogen peroxide. The sections were then incubated with anti-MV-nucleocapsid (N) protein pAb, which was produced in our laboratory^33^, and secondary antibody (Envision HRP kit; Dako). Positive reactions were identified by incubating them with DAB reaction solution. As a negative control, species-matched and filtered sera were used instead of primary antibodies. Images were captured with a Nikon microscope (Nikon, Melville, NY). The sections were fixed for fluorescence microscopy in acetone and stained with Hoechst 33342 (Cambrex Bio Science Walkersville Inc., Walkersville, MD) diluted 1:10,000, and were mounted in Dako Glycerol Mounting Medium. Images were taken on an FV1000 microscope.

### Cell sorting

SW48 cells were stained with anti-nectin-4 mAb and Alexa-Fluor-488-conjugated anti-mouse IgG Ab. Single cells were identified with forward scatter (FSC)-height/FSC-width (FSC-H/FSC-W) and 7-AAD gates, and the nectin-4-positive and -negative cells were then sorted with an SH800 cell sorter (Sony, Tokyo, Japan).

### Statistical analysis

Two-tailed Welch’s *t*-test were used for the statistical analysis of the data, and *p* values of <0.05 were considered statistically significant.

## Additional Information

**How to cite this article**: Amagai, Y. *et al*. Oncolytic Activity of a Recombinant Measles Virus, Blind to Signaling Lymphocyte Activation Molecule, Against Colorectal Cancer Cells. *Sci. Rep*. **6**, 24572; doi: 10.1038/srep24572 (2016).

## Figures and Tables

**Table 1:** Mutational status of the *KRAS*, *BRAF*, and *PIK3CA* genes in each cell line used in this study.

	ATCC No.	Mutations		
		KRAS	BRAF	PIK3CA
CaCo-2	CRL-2102	WT	WT	WT
DLD1	CCL-221	G13D	WT	E545K
HT29	HTB-38	WT	V600E	WT
LS174T	CL-188	G12D	WT	H1047R
SW48	CCL-231	WT	WT	WT
SW948	CCL-237	WT	WT	E542K
HCT116	CCL-247	G13D	WT	H1047R
LoVo	CCL-229	G13D	WT	WT
RKO	CRL-2577	WT	V600E	H1047R
SW480	CCL-228	G12V	WT	WT

WT, wild type. The mutation patterns in each cell line are indicated according to the references^29^,^30^.
